# Population Genetics of Manila Clam (*Ruditapes philippinarum*) in China Inferred from Microsatellite Markers

**DOI:** 10.3390/biology12040557

**Published:** 2023-04-06

**Authors:** Sichen Zheng, Tianshi Zhang, Kang Tu, Li Li, Zhihong Liu, Biao Wu, Liqing Zhou, Xiujun Sun

**Affiliations:** 1 National Key Laboratory of Mariculture Biobreeding and Sustainable Goods, Yellow Sea Fisheries Research Institute, Chinese Academy of Fishery Sciences, Qingdao 266071, China; 2 College of Fisheries and Life Science, Shanghai Ocean University, Shanghai 201306, China; 3 Laboratory for Marine Fisheries Science and Food Production Processes, Pilot National Laboratory for Marine Science and Technology, Qingdao 266237, China; 4 Putian Institute of Aquaculture Science of Fujian Province, Putian 351100, China; 5 National Oceanographic Center, Marine Science Research Institute of Shandong Province, Qingdao 266104, China

**Keywords:** *Ruditapes philippinarum*, SSR, genetic diversity, genetic differentiation, effective population size

## Abstract

**Simple Summary:**

The Manila clam (*Ruditapes philippinarum*) is one of the most commercially important bivalves along the coast of China. The increasing expanding of clam culture may result in some serious problems. In this paper, we investigated the genetic diversity and differentiation of *R. philippinarum* populations and tested the hypothesis that clam population differentiation is influenced by the southern breeding and northern culture. The present findings will provide useful information for natural resource conservation and genetic breeding of the Manila clam in China.

**Abstract:**

The Manila clam (*Ruditapes philippinarum*) is one of the most commercially important bivalves along the coast of China. With the continuous expansion of clam farming scale, it may lead to some serious problems, including loss of genetic variation, inbreeding depression, and reduced effective population size (N_e_). In the present study, eleven microsatellite markers were used to investigate the genetic diversity and differentiation among 13 clam populations along the coast of China. As a result, 150 alleles were detected according to the genotyping results of eleven microsatellite loci. The observed heterozygosity (H_o_) was estimated to be ranging from 0.437 to 0.678, while the expected heterozygosity (H_e_) was calculated to be varying from 0.587 to 0.700. F_st_ values between populations ranged from 0.0046-0.1983. In particular, the Laizhou population had the highest genetic variability, which was significantly different from the others (all F_st_ values > 0.1). For all the clam populations, there was no significant linear regression between genetic and geographic distance, indicating that these populations do not follow a pattern of isolation by distance (IBD). Genetic structure was estimated according to NJ, principal coordinates (PCoA), and structure-based clustering. Estimates of effective population size range from dozens to thousands among different populations, based on linkage-disequilibrium and molecular coancestry methods. The results reveal the genetic diversity of clams and verify the hypothesis that clam population differentiation may be influenced by the mode of southern breeding and northern culture, providing guiding information for natural resource conservation and genetic breeding of clams.

## 1. Introduction

The Manila clam (*Ruditapes philippinarum*) is an important marine bivalve living in the intertidal zone and has the second largest production among bivalve mollusks [1]. In China, it is widely distributed in the coastal areas from Liaoning in the north to Hainan in the south [2]. It has become one of the most commercially important bivalves in the shellfish industry, with an annual production of more than three million tons [3]. In recent years, more than 60% of adult clams are produced in Liaoning and Shandong provinces [3]. In contrast, clam seeds for culture in northern China are mainly purchased from the artificial breeding in southern China. The mode of southern breeding and northern culture may have some negative impacts in local populations such as loss of genetic variation, inbreeding depression, and reduced effective population size [2,4]. Artificial breeding with a small number of parents may increase the probability of cross-generation inbreeding depression, possibly decreasing their ability to adapt to new and challenging environments. [5]. However, the current genetic structure in a wide range of clam populations remains largely unknown. Therefore, it is essential to investigate the genetic diversity and differentiation of clam populations along the coast of China.

Genetic variation can affect the ability of aquatic animals to adapt to environmental changes [6]. Examination of genetic variation is critically important for the suitable management and conservation of natural and cultured populations in aquatic animals [7]. Molecular genetic markers are powerful tools to detect genetic variation among populations in fisheries [8]. Among the available molecular markers, microsatellite or simple sequence repeat (SSR) markers have been widely accepted as the popular molecular tools in population genetics and parentage analyses due to their high polymorphism and codominance [9]. For instance, the application of SSR in population genetics has been reported in a variety of aquatic animals, such as pearl mussel (*Hyriopsis cumingii*), ridgetail white prawn *(Exopalaemon carinicauda*), Silond catfish *(Silonia silondia*), Pacific abalone (*Haliotis Discus hannai)*, blood clam (*Barbatia virescens*)*,* and crab *(Portunus trituberculatus)* [10,11,12,13,14,15]. Despite this, most of these microsatellite studies are relying on the traditional silver staining of DNA in polyacrylamide gels, which may cause some typical sources of scoring errors capable of biasing biological conclusions, such as stuttering and null alleles [16]. SSRs are also limited by the relatively low-throughput genotyping because of their difficulties for automation and data management compared with SNPs. Despite this, SSRs can be accomplished through co-amplification of multiple microsatellites in a single PCR cocktail by multiplexing, which has been improved by decreasing genotyping costs and increasing throughput, e.g., using labelled M13-tails [17,18,19]. However, the current practices of multiplexing microsatellites in population genetics are lagging, especially in mollusks.

In this study, the new multiplex SSR method has been performed by using labelled M13-tails, providing a cost-effective method for SSR genotyping in clams. Eleven polymorphic microsatellite markers were selected to analyze the genetic diversity and differentiation of the Manila clam (*R. philippinarum*) along the coast of China. The examination of population genetic structure and differentiation of the clams aims to verify the hypothesis that clam population differentiation may be affected by the mode of southern breeding and northern culture. The present findings will not only provide useful information for genetic structure in a wide range of populations, but also help to promote natural conservation and genetic breeding of clam *R. philippinarum*.

## 2. Materials and Methods

### 2.1. Sample Collection and DNA Extraction

**Figure 1:**
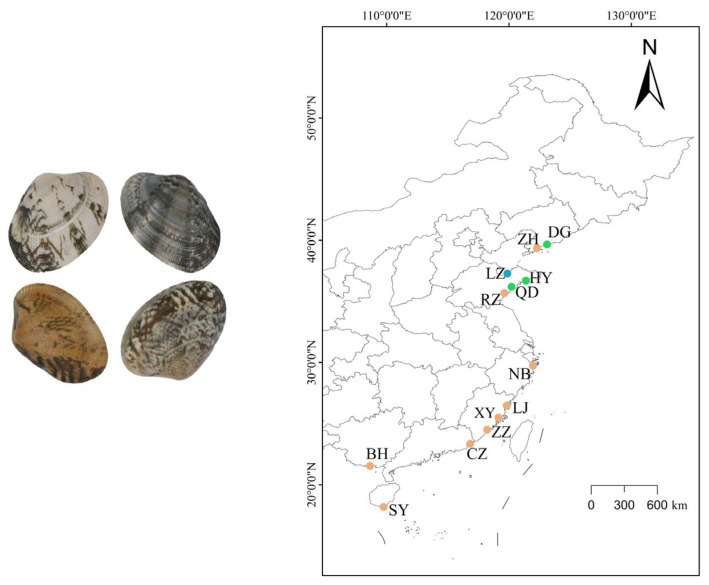
The sampled location for the Manila clam (*Ruditapes philippinarum*) along the coast of China. The map of the People’s Republic of China is downloaded from the website of http://bzdt.ch.mnr.gov.cn/, accessed on 7 December 2021. The color dots representing the three groups revealed by the PCoA analysis: blue (LZ), green (DG, HY, QD), and orange (ZH, RZ, NB, LJ, XY, ZZ, CZ, BH, and SY).

A total of 406 clams (*R. philippinarum*) were collected from the northern and southern coast of China (Figure 1). The sampling time, locations, and quantities for the clam samples are summarized in Table 1. Six populations were collected from the southern coast, including Chaozhou (CZ), Lianjiang (LJ), Ningbo (NB), Sanya (SY), Zhangzhou (ZZ), and Beihai (BH). Meanwhile, six populations were collected from the northern coast, including Laizhou (LZ), Rizhao (RZ), Qingdao (QD), Haiyang (HY), Donggang (DG), and Zhuanghe (ZH). In addition, the sample of XY was collected from the selected clam population for rapid growth. For each sample, the foot muscle of clams was dissected and preserved in 100% ethanol. The traditional phenol chloroform method was used for DNA extraction from the foot muscle. After DNA extraction, the quality of DNA was assessed by 1.5% agarose gel electrophoresis. The DNA concentration was measure by the Nanodrop Lite ultra-micro spectrophotometer. All the DNA samples were diluted into 50 ng/µL and stored at −20 °C.

### 2.2. Primer Screening and PCR Amplification

Eleven pairs of microsatellite markers with stable amplification were selected from the previous reports [20,21]. The basic information for primer sequences and PCR conditions is shown in Table 2. The fluorescent labeling for SSRs using M13 tails were performed according to the previous study with minor modifications [17]. Briefly, three primers were used for each PCR amplification: (1) the first one was a forward primer with M13 tails at the 5′ end; (2) the second one was an SSR reverse primer; (3) the third one was an M13 universal primer with a fluorescent label (the 5′ end labeled with 6-carboxy-fluorescine (Fam), hexachloro-6-carboxy-fluorescine (Hex), 6-carboxy-X-rhodamine (Rox), and tetramethylrhodamine (Tamra) fluorescent groups). The selected primer pairs were sorted according to the size ranges. The similar size fragments were labeled with different fluorescence, while different size fragments were labeled with the same fluorescence (Figure S1, Table 2). The PCR reaction system included template DNA 50 ng, 2× Taq plus Master Mix II 8 µL (Nanjing Vazyme Biotechnology Co., Ltd., Nanjing, China), forward primer 0.04 µL (10 µmol/L), reverse primer 0.16 µL (10 µmol/L), and fluorescent labeled M13 primer 0.16 µL (10 µmol/L), plus dd H_2_O to 16 µL. The PCR reactions were performed as follows: 94 °C for 5 min; 30 cycles of 94 °C for 30 s, 53 °C for 45 s, and 72 °C for 45 s; 8 cycles of 94 °C 30 s, 53 °C 45 s, 72 °C 45 s; a final extension at 72 °C for 10 min. The quality of PCR products was detected by 1.5% agarose gel electrophoresis. Finally, 1 µL of PCR products was added to 22 µL formamide and 0.5 µL ROX standard and run on the ABI 3730XL (Shanghai Sangon Bioengineering Co., Ltd., Shanghai, China).

### 2.3. Data Processing

The software MSAnalyzer 4.05 was used to calculate number of alleles (N), the observed heterozygosity (H_o_), and the expected heterozygosity (H_e_) [22]. The allelic richness (A_r_) and inbreeding coefficient (F_is_) were calculated through FSTAT 2.9.3 [23]. The significant positive F_is_ values indicate inbreeding within populations (excess of homozygotes), whereas the significant negative F_is_ values represent an excess of heterozygosity. The differences of allelic richness among different groups were compared by the Kruskal–Wallis test of SPSS 26. Furthermore, differences in the allelic richness for each population at each locus was tested using a Kruskal–Wallis rank sum analysis [24]. Hardy–Weinberg equilibrium test and genetic differentiation coefficient (F_st_) were calculated by Genepop 4.0 [25]. For the STRUCTURE analysis, the optimal K value was calculated according to the procedure of Evanno [26], and then the Q value corresponding to the optimal K value was obtained through the repeated sampling analysis of the structure operation results by the CLUMPP software [27]. The genetic structure figure of 13 populations were finally constructed by the software distruct1.1. Genetic distance (Ds) was calculated based on POPULATION software, and then MEGA X was used to build NJ and ME evolutionary trees [28]. An analysis of molecular variance (AMOVA) was performed by the ARLEQUIN program ver. 3.0 to measure the components of variance among and within the populations [29]. A principal component analysis (PCoA) was performed based on the covariance matrix of allele frequencies using GenAlEx 6.3. Mantel test was also performed with GenAlEx 6.3. Linkage-disequilibrium (LD) and molecular coancestry (Cn) methods were used to estimate Ne by using LDNe and NeEstimator v2.0 [30,31].

## 3. Results

### 3.1. Genetic Diversity within Populations

For the eleven microsatellite loci, the genotyping results of 408 individuals were derived from 13 clam populations with sample sizes ranging from 24 to 40. The descriptive genetic statistics (e.g., N, A_r_, G_D_, H_o_, and H_e_) were shown for each locus and population in Table S1. As a result, more than nine alleles were found in each of the eleven microsatellite loci, with the maximum alleles (18 alleles) detected in Rp-03. The mean allelic richness (A_r_) varied from 3.2 (Rpt100 and Rp-07) to 5.5 (Rpt106). At the population level, the average of observed heterozygosity (H_o_) was calculated to be ranging from 0.437 to 0.678, while the expected heterozygosity (H_e_) was estimated to be varying from 0.587 to 0.700. Among the eleven loci, the highest H_e_ value (0.700) was detected in the selected population of XY, while the lowest value (0.587) was found in the QD population. The number of alleles per locus in each population ranged from 2 to 11, and allelic richness per locus varied from 1.7 to 5.5. For all these populations, the LZ population had the largest number of alleles (6.9), as well as the maximum of allele richness (4.1). In contrast, the least number of alleles (5.1) and the minimum of allele richness (3.3) were found in the ZH population. Despite this, no significant difference in allelic richness was detected among these populations (Kruskal–Wallis test, *p* > 0.05). The positive values of F_is_ were consistently found in all the populations, except for HY population (Table S1). A total of 69 (48.3%) of the 143 locus–population combinations were significantly deviant from Hardy–Weinberg equilibrium (HWE) after the Bonferroni correction (*p* < 0.005).

### 3.2. Genetic Differentiation among Populations

**Figure 2:**
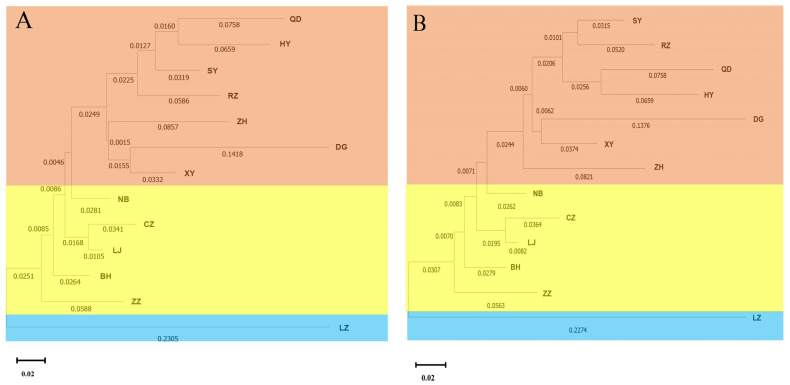
Cluster analysis of 13 populations of *R. philippinarum* by NJ (the neighbor-joining) and ME (the minimum evolution) methods. (**A**) NJ tree; (**B**) ME tree. The different colors are used to differentiate the clustered clades among the clam populations. The orange color represents the independent branch of the clam populations, including QD, HY, SY, RZ, ZH, DG, and XY. The orange clade clusters with the yellow branches (NB, CZ, LJ, BH, and ZZ), forming into a higher-level clade. The blue color represents the independent branch for the LZ population different from the large clade.

Pairwise F_st_ values among the 13 populations were shown in Table 3. Pairwise F_st_ values across all samples were ranging from 0.0046 to 0.1983 (Table 3). The lowest genetic differentiation was detected between population LJ and NB (F_st_ = 0.0046, *p* < 0.01), whereas the highest differentiation was found between the QD and LZ populations (F_st_ = 0.1983, *p* < 0.01). The genetic differentiation between the LZ population and other populations is relatively high, varying from 0.1020 to 0.1983 (*p* < 0.01). The genetic distances (D_S_) among populations were also displayed in Table 3. The lowest genetic distance (0.0446) was detected between CZ and LJ, while the largest value (0.4702) was found between LZ and DG. The genetic distances between LZ and the other 12 populations were ranging from 0.1141 to 0.4702. The NJ and ME clustered dendrograms were constructed based on the pairwise genetic distances (Figure 2). As illustrated, no obvious pattern of genetic differentiation was detected among the populations from the northern and southern coast. As displayed in Figure 2A, three northern populations (QD, HY, and RZ) and one southern population (SY) were clustered into one independent branch. In the meantime, two northern populations (ZH and DG) and the selected population (XY) were clustered into another branch. Subsequently, the two small branches were merged with some southern populations (NB, CZ, LJ, BH, and ZZ). The large branch was finally clustered with the LZ population. The clustering result of the ME tree is similar to the NJ tree.

**Figure 3:**
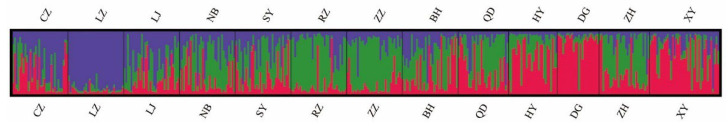
Estimated genetic clusters of thirteen *R. philippinarum* populations. The graph is based on the proportion of individuals per population in the inferred clusters according to STRUCTURE. Each of the three colors represents a different genetic cluster, and black lines separate the populations.

For each locus, the F_st_ value was ranging from 0.0357 to 0.1729 (*p* < 0.01), with an average of 0.0663 (Table S2). The N_m_ value of gene flow was varying from 1.1958 to 6.6869, with an average of 4.5900. The F_is_ value for each locus was calculated to be ranging from −0.1021 to 0.6411, with an average of 0.1855. The STRUCTURE analysis revealed K = 3 was the most probable number of populations to explain the observed genotypes (Figure 3). As indicated by STRUCTURE analysis, all the individuals can be divided into three subgroups (Blue, Green, and Red; Figure 3). Consistently, the individuals from each population were also classified into the three genetic clusters, suggesting the high gene flow of these clam populations. According to AMOVA analysis, the greatest number of variances occurred within individuals (67.33%), compared to 25.7% among individuals and 6.97% among the populations (Table 4).

**Figure 4:**
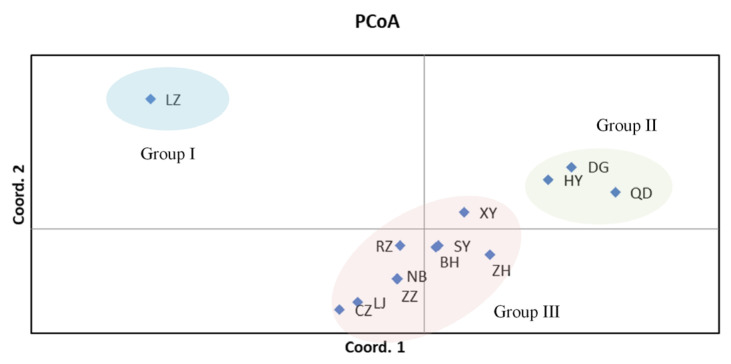
Principal coordinate analysis of genetic similarity among 13 clam populations.

**Figure 5:**
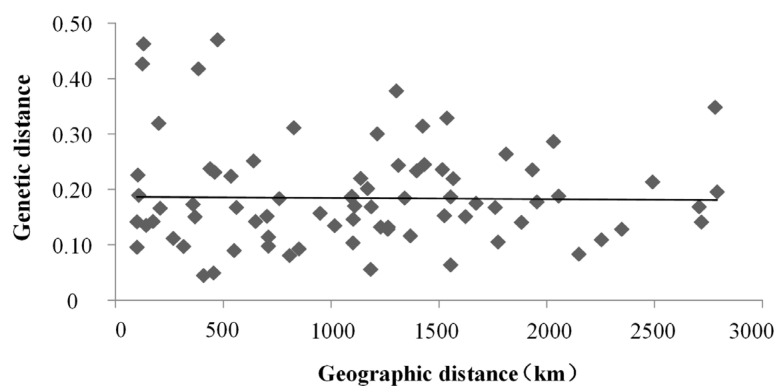
The non-significant linear regression between genetic distance and geographic distance based on eleven microsatellite loci in the clam samples (Y = −5.41 × 10^−6^X + 0.1959, R^2^ = 0.0018, 10,000 permutations).

The visual representation of genetic distances among the 13 populations revealed by PCoA analysis was displayed in Figure 4. In accordance with STRUCTURE results, PCoA analysis indicated that these 13 populations were mainly formed into three main groups: group I (LZ), group II (HY, DG, and QD), and group III (XY, SY, ZH, BH, RZ, NB, ZZ, LJ, and CZ) (Figure 4). A plot of the first and second principal coordinates is presented, accounting for 37.63% and 19.25% of the total variation, respectively. Samples from group I were well-differentiated from others on the first and second axes, while samples from group II were mainly separated on the first axis. Although four northern sites (Group I and Group II) seem to be different from other sites (Figure 4), the genetic difference within northern populations (Group I and Group II) are much greater than the difference between Group II (northern populations) and Group III (comprises both northern and southern populations). Based on the Mantel tests in GenAlex6.51, no significant linear relationship was detected between genetic distance and geographic distance in the clam samples (Y = −5.41 × 10^−6^X + 0.1959, R^2^ = 0.0018, *p* > 0.05; Figure 5). The results indicate that the clam populations do not follow a pattern of isolation by distance (IBD; Figure 5), and this is evidenced by high gene flow among populations within the large geographic scales (Group III, Figure 4). For instance, ZH and BH are thousands of kilometers apart (>2500 km), but they have a relatively low genetic differentiation level, F_st_ = 0.058.

### 3.3. Estimation of Effective Population Size (N_e_)

Two single-sample methods were used to estimate N_e_ for all 13 samples collected in Table 5. The LDNe method yielded part negative N_e_ estimates (Table 5). According to the LD and Cn methods, the N_e_ values of most populations were low except for the XY population (N_e_ = 375.4). The lowest values of N_e_ were found in Chaozhou and Donggang, having extremely low N_e_ of less than the critical value (N_e_ = 50). Generally, the N_e_ values estimated from the Cn method were relatively lower than those from the LD method.

## 4. Discussion

### 4.1. Genetic Diversity of Manila Clams in Different Populations from North to South

High levels of genetic diversity appear to be a common feature of marine bivalves [32]. In this study, microsatellite analysis of *R. philippinarum* populations revealed a relatively higher level of genetic diversity (H_e_ = 0.636) than those estimates from allozymic analysis and other DNA-based analyses, such as mtDNAs, AFLP, and RAPD [4,33,34]. Consistent with our study, high levels of genetic diversity estimated from microsatellite markers were also observed in other bivalves, such as *Crassostrea gasar* (H_e_ = 0.843 [35], *Barbatia virescens* (H_e_ = 0.790 [9]), and *Crassostrea ariakensis* (H_e_ = 0.805 [36]). Large population sizes and high nucleotide mutation rates are likely to be the major contributors to the high levels of genetic diversity estimated from microsatellites [37,38].

Departures from Hardy–Weinberg equilibrium (HWE) were measured through the significance of permutation tests for the null hypothesis, F_is_ = 0 [39]. In the present study, the significant heterozygote deficiency was detected in clam populations according to these genotyped microsatellite loci (F_is_ = 0.1855; *p* < 0.05). In addition to clams, multi-locus heterozygosity deficiencies have been previously widely reported in many other bivalves [40,41]. Early explanation for the departure from HWE in bivalves mainly involved null alleles, natural selection, inbreeding, and Wahlund effects [42]. However, the recent hypothesis of genetic load shows more compelling evidence for this phenomenon, indicating the large genetic load of partially dominant or additive detrimental mutations in wild adult populations [43,44]. It is therefore suggested that the high genetic load is largely responsible for heterozygote deficits in wild populations and segregation distortion in pair crosses, resulting in substantial genetic sterility [44]. Further studies will be needed to elucidate the genetic load by pair crosses of clams.

### 4.2. The Genetic Differentiation among Clam Populations

The overall genetic differentiation among these populations was moderate but highly significant (global F_st_ = 0.066, *p* < 0.001),indicating the existence of the genetic heterogeneity among populations. As the wild population in Laizhou Bay (Shandong province, North China), the LZ population remains the population with the highest level of genetic variability, showing great differentiation with other populations. This is consistent with the previous studies, supporting the natural status of clam populations with high levels of genetic variability [2,45]. As reported, natural selection continuously removes neutral diversity linked to either beneficial or deleterious variants [46]. In contrast to the LZ populations, other clam populations are likely to have low differentiation and high glow flow according to cluster and PCoA analysis. In the present study, the low differentiation among different populations supports the hypothesis that the genetic structure of clams may be influenced by the mode of southern breeding and northern culture.

In this study, the clam populations do not follow a pattern of isolation by distance, and this contrasts with the reported IBD pattern caused by larval dispersal in other coastal bivalve species [41]. For the clam populations, high gene flow among populations so far apart seems unlikely to be caused by the larval dispersal. The more reasonable explanation for this is probably due to seeds’ transplantation by local farmers among different culture regions. In recent decades, there is considerable translocation of clam seeds cultivated in Fujian province (south) to culture sites in Shandong and Liaoning provinces (north) [4,20,21]. Therefore, the artificial breeding and culture of clams may increase the gene flow of clams, resulting in the low genetic differentiation between northern and southern populations, as evidenced by our present results. The low genetic differentiation between northern and southern populations has also been detected in the previous studies [2,47]. Therefore, the present findings do not support the typical pattern of genetic differentiation between northern and southern populations due to geographic isolation. The translocation of clam seeds may be served as one of the major factors influencing the population genetic structure of the clams. Adapted conservation measures for wild populations are required to maintain high levels of genetic diversity of clams on the coast of China. In order to protect the wild clam populations, it is necessary to take measures to prevent excessive harvesting and formulate laws and regulations to limit the number and time for clam harvesting. It is also important to ensure that natural habitats of clams have not been occupied or damaged by environmental pollution. We recommend the use of responsible conservation aquaculture protocols, such as large numbers of local adult clams for bloodstocks and new techniques reducing hatchery selection to facilitate the management of genetic variability [46]. However, simply increasing the number of breeders does not necessarily increase the effective breeding numbers in shellfish hatcheries. Therefore, the development of breeding strategies and optimization of production is also important in the maintaining of genetic diversity, such as pedigree monitoring by genetic markers and performing controlled spawning [47,48,49]. Recently, the rapid development of high-throughput sequencing methods have facilitated the incorporation of genomic tools in clam breeding programs by control parental contribution [49]. Overall, these strategies are recommended for the retention of high genetic variability in clam *R. philippinarum*, especially for the wild population in Laizhou Bay.

### 4.3. Estimation of Effective Population Sizes in Clam Populations

The effective population size (N_e_), a key parameter in evolutionary biology, determines the rates of genetic drift and loss of genetic variability and modulates the effectiveness of selection [50]. For wild populations, the supplement with artificially breeding individuals can lead to the N_e_ reduction, known as the Ryman–Laikre effect [51]. As reported, the reduction of N_e_ would lead to a collapse of local genetic adaptation, which could expose local populations to adverse effects [52,53,54].

The previous studies have indicated that N_e_ of shellfish bloodstocks should be large enough to produce the first generation with relatively medium or high genetic diversity [55]. The small N_e_ population will lead to the depletion of rare alleles, increasing of the random drift of the original population, and thus threatening of the sustainability of populations [56]. As a rule-of-thumb in populations, N_e_ in the short term should not be less than 50, and in the long term should not be less than 500 [57]. The estimates of N_e_ thresholds for avoiding inbreeding depression (N_e_ = 50) and retention of genetic variation for future adaptations (N_e_ = 500) can be used as a guiding principle to indicate the short- and long-term genetic viability of populations [55,56].

In the present study, small N_e_ values (less than 50) have been obtained in several populations (e.g., QD and CZ) according to LD and Cn methods. The small N_e_ values may be caused by inadvertent selection of the best offspring produced by a few parents and asymmetric reproduction [58]. Despite this, the accumulation of inbreeding might have some negative effects on survival rates of clams in these populations with small N_e_ values [21,22]. Thus, it is essential to recover the local populations by the conservation programs (e.g., broodstock management and controlled spawning) to maintain a minimum viable population to maintain the evolutionary potential [59,60]. Surprisingly, negative N_e_ values from the LD method have been detected in multiple populations of clams, probably due to the linkage disequilibrium generated by the sampling process and inadequate correction [61]. If N_e_ is very large or limited data are available, by chance ${}^{r}$^2^ (mean squared correlation of allelic frequencies) can be smaller than the sample size correction, resulting in the negative estimates of N_e_ [58,62]. Therefore, the negative estimates may occur when genetic results can be explained entirely by sampling error without invoking any genetic drift, interpreted as the infinite N_e_ [58]. This is also supported by the computer simulations, indicating that the LD method is biased when the sample size is small (<100) and below the true N_e_ [61]. Despite this, the lower bound of CIs in this study can provide some useful information for the plausible limits of these negative N_e_ values. The future estimation of N_e_ needs an extensive evaluation in larger sample sizes using increased numbers of loci and alleles. Despite uncertainties related to the small sampling size, N_e_ estimates obtained by the two applied methods can provide useful complementary information for conservation programs to prevent inbreeding depression and loss of genetic variation. According to the present findings, the small N_e_ values, as well as the low differentiation, may be caused by few broodstock used in southern hatcheries, with offspring transferred to the northern coast for culture at the mode of southern breeding and northern culture.

## 5. Conclusions

In this study, genetic diversity and differentiation were investigated by 11 microsatellite loci for *R. philippinarum* (Manila clam) populations from the coastal areas of China. The multiplex PCR using the labelled M13-tails was shown to be a cost-effective method for SSR genotyping in clams and mollusks, provided that the sufficient sampling size is ensured. The present findings support that the genetic population structure of clams may be influenced by the mode of southern breeding and northern culture. The assessment of the genetic diversity of *R. philippinarum* populations is of considerable importance for the optimal development of programs aimed at the conservation of cultivated and wild genotypes in the ecosystems. The present findings will provide guiding information on natural resource conservation and genetic breeding of the Manila clam in China. The highest level of genetic variability and greatest differentiation with other populations was confirmed for the wild Laizhou population. It was suggested that multi-locus heterozygote deficiency and segregation distortion in such populations may be caused by high genetic load. No relation was found between genetic and geographic distance, implying clam aquaculture may be served as one of the major factors influencing clam population genetic structure. Despite uncertainties related to the small sampling size, N_e_ estimates obtained by the applied methods can provide useful complementary information for conservation programs to warn about inbreeding depression and loss of genetic variation, thereby serving the needs of natural resource conservation.

## Figures and Tables

**Table 1:** Sample code, location, collection date, and sample sizes for all populations of *R. philippinarum*.

Sample Code	Name	Location	Collection Date	Sample Size
CZ	Chaozhou	Chaozhou, Guangdong Province	July 2020	32
LZ	Laizhou	Laizhou, Shandong Province	August 2020	32
LJ	Lianjiang	Lianjiang, Fujian Province	July 2020	32
NB	Ningbo	Ningbo, Zhejiang Province	July 2020	32
SY	Sanya	Sanya, Hainan Province	June 2020	32
RZ	Rizhao	Rizhao, Shandong Province	August 2020	32
ZZ	Zhangzhou	Zhangzhou, Fujian Province	July 2020	32
BH	Beihai	Beihai, Guangxi Province	June 2020	32
QD	Qingdao	Qingdao, Shandong Province	August 2020	29
HY	Haiyang	Haiyang, Shandong Province	August 2020	28
DG	Donggang	Donggang, Liaoning Province	August 2020	24
ZH	Zhuanghe	Zhuanghe, Liaoning Province	August 2020	29
XY	Selected population	Putian, Fujian Province	August 2020	40

**Table 2:** Primer sequences and information about microsatellite loci from *Ruditapes philippinarum*.

Sequence	Locus	Accession	Primer (5′-3′)	Repeat Unit	Tm (°C)	Fluorescent Labelling	Size Ranges/ bp
1	Rpt23	KC811247	F: AGCGTGTTGCTGCTCTTC	(AGC)6	48	FAM	81–117
			R: ATTACTCCCACTGTTCGT				
2	Rp-07	AM874000	F: TATGGCTGGTTTGGACTG	(AT)7	51	TAM	119–151
			R: TCCCGTTACACTTACTTTCA				
3	Asari16	AB257421	F: GCTCGAGTCTGATTGGCTACTGAA	(CT)12	55	ROX	151–174
			R: GGTATCTAGTCAGCTCTTGCAGTA				
4	Rp-03	AM873616	F: CCGCTGTGAGGAGACCAA	(TTG)6	58	FAM	170–213
			R: CCGCCTATGTGACAAAATGA				
5	Rpt36	KC811251	F: TTGAGGCATCAATAACTTTC	(TTG)8	50	TAM	230–268
			R: ACTTCTGCATCTCGGCTA				
6	Rpt100	KC811260	F: TCATTTCCAAGGCAGGTA	(ATG)5	50	ROX	237–274
			R: GAGGTGTTGAAGGAGCAG				
7	Rpt106	KC811263	F: ACCTCAGTTCAAATGTCT	(AGT)6	48	HEX	373–409
			R: AATACTAACGCTGTGGAT				
8	Rpt105	KC811262	F: GGTATGGTGGTAAATGGA	(GTT)5	46	FAM	375–411
			R: TCATAGGTAGGGTGGTTT				
9	Rpt67	KC811255	F: GGGTTCTTCTGTAGTTGG	(GAA)5	46	TAM	379–415
			R: TGAGAAATCAGACCCAAT				
10	Rpt32	KC811249	F: TCACTTTCTGCTCCTACA	(CAT)5	47	ROX	415–451
			R: AAAGGGAATCTCGTGGTG				
11	Rpt83	KC811257	F: GGTCGCCTAATTTCGTAG	(TGT)7	46	HEX	429–472
			R: TAATAATTTTCCTGGAGCTCTGGCG				

**Table 3:** Pairwise F_st_ (under diagonal) and Nei’s genetic distance Ds (above diagonal) of *R. philippinarum*.

Population	CZ	LZ	LJ	NB	SY	RZ	ZZ	BH	QD	HY	DG	ZH	XY
CZ	0	0.3291	0.0446	0.0927	0.1573	0.1847	0.1353	0.0810	0.2450	0.2361	0.2355	0.1407	0.0973
LZ	0.1084 **	0	0.3005	0.3113	0.3484	0.3194	0.3144	0.2863	0.4629	0.4265	0.4702	0.4178	0.3777
LJ	0.0048	0.1020 **	0	0.0492	0.1161	0.1349	0.1117	0.0556	0.1863	0.2015	0.2198	0.1525	0.0956
NB	0.0270 **	0.1021 **	0.0046	0	0.1050	0.1422	0.1141	0.0639	0.1517	0.1835	0.2201	0.1461	0.0899
SY	0.0474 **	0.1089 **	0.0285 **	0.0227 **	0	0.0835	0.1874	0.1731	0.1091	0.1284	0.1954	0.1685	0.1320
RZ	0.0648 **	0.1065 **	0.0435 **	0.0434 **	0.0260 **	0	0.1324	0.1868	0.1898	0.1660	0.2514	0.1676	0.1701
ZZ	0.0561 **	0.1181 **	0.0423 **	0.0375 **	0.0593 **	0.0484 **	0	0.0979	0.2433	0.2337	0.2641	0.1674	0.1423
BH	0.0425 **	0.1084 **	0.0252 **	0.0145 **	0.0348 **	0.0654 **	0.0413 **	0	0.1775	0.1881	0.2136	0.1410	0.1035
QD	0.1349 **	0.1983 **	0.1157 **	0.0857 **	0.0809 **	0.1048 **	0.1224 **	0.0794 **	0	0.1417	0.2242	0.2310	0.1688
HY	0.0982 **	0.1453 **	0.0864 **	0.0725 **	0.0488 **	0.0773 **	0.0904 **	0.0478 **	0.0679 **	0	0.2373	0.1507	0.1284
DG	0.1058 **	0.1653 **	0.0947 **	0.0827 **	0.0716 **	0.0977 **	0.1060 **	0.0716 **	0.0742 **	0.0559 **	0	0.2263	0.1750
ZH	0.0610 **	0.1444 **	0.0612 **	0.0510 **	0.0519 **	0.0599 **	0.0679 **	0.0580 **	0.0933 **	0.0508 **	0.0801 **	0	0.1510
XY	0.0415 **	0.1096 **	0.0405 **	0.0290 **	0.0410 **	0.0561 **	0.0557 **	0.0332 **	0.0602 **	0.0427 **	0.0510 **	0.0490 **	0

Note: “**” indicates the F_st_ reaches significant level at *p <* 0.01.

**Table 4:** Analysis of molecular variance (AMOVA) in thirteen populations of *R. philippinarum*.

Source of Variation	d.f.	Sum of Squares	MS	Est. Var.	Percentage Variation
Among Populations	12	260.237	21.686	0.273	6.97%
Among Individuals	393	1828.026	4.651	1.007	25.70%
Within Individuals	406	1071.000	2.638	2.638	67.33%
Total	811	3159.262		3.918	100.00%

Degree of freedom (d.f.), mean square (MS), variance component (Est. Var.).

**Table 5:** Effective population sizes (N_e_) for *R. philippinarum* populations estimated by the linkage disequilibrium (LD) and molecular coancestry (Cn) methods.

	LD				Cn		
Pop	n	$:{\hat{r}}^{2}$	$E\;(:{\hat{r}}^{2})$	N_e_ (95% CI)	Pop	n	N_e_ (95% CI)
CZ	28.5	0.0483	0.0391	31.7 (20.3–58.8)	CZ	30.1	7.2 (3.4–12.5)
LZ	31.0	0.0384	0.0355	114.9 (50.1–Infinite)	LZ	31.5	Infinite (Infinite–Infinite)
LJ	29.5	0.0373	0.0377	−876.7 (92.3–Infinite)	LJ	30.8	Infinite (Infinite–Infinite)
NB	28.8	0.0375	0.0387	−258.6 (129.9–Infinite)	NB	30.4	32.8 (0–164.9)
SY	28.6	0.0434	0.0390	68.2 (33.3–510.2)	SY	29.8	20.7 (1.5–64.4)
RZ	29.0	0.0418	0.0384	88.3 (36.7–Infinite)	RZ	30.5	18.9 (0–94.8)
ZZ	29.2	0.0374	0.0380	−513.7 (74.7–Infinite)	ZZ	30.3	Infinite (Infinite–Infinite)
BH	24.7	0.0419	0.0458	−79.9 (290.4–Infinite)	BH	27.9	54.3 (0.1–272.7)
QD	17.7	0.0526	0.0672	22.7 (-39.9–Infinite)	QD	19.0	11.9 (2–30.6)
HY	18.2	0.0526	0.0649	−26.5 (-47.9–Infinite)	HY	22.4	11.1 (2.7–25.4)
DG	14.9	0.0776	0.0827	−62.1 (58.3–Infinite)	DG	19.5	6.2 (4.2–8.5)
ZH	22.9	0.0497	0.0498	−4158.7 (47.7–Infinite)	ZH	26.5	17.0 (2.8–43.7)
XY	30.3	0.0374	0.0365	375.4 (73.5–Infinite)	XY	34.8	Infinite (Infinite–Infinite)

Mean sample sizes per locus (n), mean squared correlation of allelic frequencies over ($:{\hat{r}}^{2}$) the expectation of $:{\hat{r}}^{2}$ based on mean sample size (E ($:{\hat{r}}^{2}$)).

## Data Availability

Not applicable.

## Supplementary Materials

The following supporting information can be downloaded at: https://www.mdpi.com/article/10.3390/biology12040557/s1, Table S1: Genetic diversity parameters of thirteen *R. philippinarum* populations. Table S2: Genetic differentiations and gene flow of thirteen *R. philippinarum* populations at different loci. Figure S1: SSR profiles generated on a capillary sequencer for a single sample. Green color, Hex fluorescence; Red color, Rox fluorescence; Blue color, Fam fluorescence; Black color, Tamra fluorescence.

## References

[1] De Montaudouin X., Lucia M., Binias C., Lassudrie M., Baudrimont M., Legeay A., Raymond N., Jude-Lemeilleur F., Lambert C., Le Goïc N. (2016). Why is Asari (=Manila) clam *Ruditapes philippinarum* fitness poor in Arcachon Bay: A meta-analysis to answer?. Estuar. Coast. Shelf Sci..

[2] Tan Y., Fang L., Qiu M., Huo Z., Yan X. (2020). Population genetics of the Manila clam (*Ruditapes philippinarum*) in East Asia. Sci. Rep..

[3] DOF (2021). China Fisheries Statistic Yearbook.

[4] Liu X., Zhenmin B., Jingjie H., Shi W., Aibin Z., Hui L., Jianguang F., Rucai W. (2007). AFLP analysis revealed differences in genetic diversity of four natural populations of Manila clam (*Ruditapes philippinarum*) in China. Acta Oceanol. Sin..

[5] Kristensen T.N., Hoffmann A.A., Pertoldi C., Stronen A.V. (2015). What can livestock breeders learn from conservation genetics and vice versa?. Front. Genet..

[6] Conover D., Clarke L., Munch S., Wagner G. (2006). Spatial and temporal scales of adaptive divergence in marine fishes and the implications for conservation. J. Fish Biol..

[7] Okumuş İ., Çiftci Y. (2003). Fish population genetics and molecular markers: II-molecular markers and their applications in fisheries and aquaculture. Turk. J. Fish. Aquat. Sci..

[8] Askari G., Shabani A., Kolangi Miandare H. (2013). Application of molecular markers in fisheries and aquaculture. Sci. J. Anim. Sci..

[9] Nie H., Niu H., Zhao L., Yang F., Yan X., Zhang G. (2015). Genetic diversity and structure of Manila clam (*Ruditapes philippinarum*) populations from Liaodong peninsula revealed by SSR markers. Biochem. Syst. Ecol..

[10] An H., Lee J., Park J. (2012). Population genetics of the Pacific abalone (*Haliotis discus hannai*) in Korea inferred from microsatellite marker analysis. Genet. Mol. Res..

[11] Bai Z., Zheng H., Lin J., Wang G., Li J. (2013). Comparative analysis of the transcriptome in tissues secreting purple and white nacre in the pearl mussel *Hyriopsis cumingii*. PLoS ONE.

[12] Mandal S., Jena J., Singh R.K., Mohindra V., Lakra W., Deshmukhe G., Pathak A., Lal K.K. (2016). De novo development and characterization of polymorphic microsatellite markers in a schilbid catfish, *Silonia silondia* (Hamilton, 1822) and their validation for population genetic studies. Mol. Biol. Rep..

[13] Liu Q., Cui F., Hu P., Yi G., Ge Y., Liu W., Yan H., Wang L., Liu H., Song J. (2018). Using of microsatellite DNA profiling to identify hatchery-reared seed and assess potential genetic risks associated with large-scale release of swimming crab *Portunus trituberculatus* in Panjin, China. Fish. Res..

[14] Wang L., Yu H., Li Q. (2019). Development of microsatellite markers and analysis of genetic diversity of *Barbatia virescens* in the southern coasts of China. Genes Genom..

[15] Li J., Li J., Chen P., Liu P., He Y. (2015). Transcriptome analysis of eyestalk and hemocytes in the ridgetail white prawn *Exopalaemon carinicauda*: Assembly, annotation and marker discovery. Mol. Biol. Rep..

[16] DeWoody J., Nason J.D., Hipkins V.D. (2006). Mitigating scoring errors in microsatellite data from wild populations. Mol. Ecol. Notes.

[17] Schuelke M. (2000). An economic method for the fluorescent labeling of PCR fragments. Nat. Biotechnol..

[18] Hayden M.J., Nguyen T.M., Waterman A., Chalmers K.J. (2008). Multiplex-ready PCR: A new method for multiplexed SSR and SNP genotyping. BMC Genom..

[19] Guichoux E., Lagache L., Wagner S., Chaumeil P., Léger P., Lepais O., Lepoittevin C., Malausa T., Revardel E., Salin F. (2011). Current trends in microsatellite genotyping. Mol. Ecol. Resour..

[20] Nie H.L.J.H., Zh, Guo W., Yan X. (2016). Analysis of genetic variability in selected lines and a wild population of R*uditapes philippinarum* using microsatellite markers. J. Fish. Sci. China.

[21] Yu Z., Yan X., Yang F., Wang J., Zhang Y., Yang F., Zhang G. (2011). Genetic diversity of different generations of the Dalian population of Manila clam *Ruditapes philippinarum* through selective breeding. Acta Ecol. Sin..

[22] Dieringer D., Schlötterer C. (2003). Microsatellite analyser (MSA): A platform independent analysis tool for large microsatellite data sets. Mol. Ecol. Notes.

[23] Goudet J. (2001). FSTAT, a Program to Estimate and Test Gene Diversities and Fixation Indices (Version 2.9.3). https://www2.unil.ch/popgen/softwares/fstat.htm.

[24] Sokal R.R., Rohlf F.J., Rohlf J.F. (1995). Biometry.

[25] Raymond M., Rousset F. (1995). An exact test for population differentiation. Evolution.

[26] Evanno G., Regnaut S., Goudet J. (2005). Detecting the number of clusters of individuals using the software STRUCTURE: A simulation study. Mol. Ecol..

[27] Jakobsson M., Rosenberg N.A. (2007). CLUMPP: A cluster matching and permutation program for dealing with label switching and multimodality in analysis of population structure. Bioinformatics.

[28] Kumar S., Stecher G., Li M., Knyaz C., Tamura K. (2018). MEGA X: Molecular evolutionary genetics analysis across computing platforms. Mol. Biol. Evol..

[29] Excoffier L., Laval G., Schneider S. (2005). Arlequin (Version 3.0): An integrated software package for population genetics data analysis. Evol. Bioinform..

[30] Waples R.S., Do C. (2008). LDNE: A program for estimating effective population size from data on linkage disequilibrium. Mol. Ecol. Resour..

[31] Do C., Waples R.S., Peel D., Macbeth G., Tillett B.J., Ovenden J.R. (2014). NeEstimator v2: Re-implementation of software for the estimation of contemporary effective population size (N_e_) from genetic data. Mol. Ecol. Resour..

[32] An H.S., Park K.J., Cho K.C., Han H.S., Myeong J.-I. (2012). Genetic structure of Korean populations of the clam *Ruditapes philippinarum* inferred from microsatellite marker analysis. Biochem. Syst. Ecol..

[33] Vargas K., Asakura Y., Ikeda M., Taniguchi N., Obata Y., Hamasaki K., Tsuchiya K., Kitada S. (2008). Allozyme variation of littleneck clam *Ruditapes philippinarum* and genetic mixture analysis of foreign clams in Ariake Sea and Shiranui Sea off Kyushu Island, Japan. Fish. Sci..

[34] Sekine Y., Yamakawa H., Takazawa S., Lin Y., Toba M. (2006). Geographic variation of the COX1 gene of the short-neck clam *Ruditapes philippinarum* in coastal regions of Japan and China. Venus.

[35] Melo M.A.D., da Silva A.R.B., Varela E.S., Sampaio I., Tagliaro C.H. (2012). Development and characterization of ten microsatellite markers for population studies of the native Brazilian oyster *Crassostrea gasar*. Conserv. Genet. Resour..

[36] Xiao J., Cordes J.F., Wang H., Guo X., Reece K.S. (2010). Population genetics of *Crassostrea ariakensis* in Asia inferred from microsatellite markers. Mar. Biol..

[37] Hoshino A.A., Bravo J.P., Nobile P.M., Morelli K.A. (2012). Microsatellites as Tools for Genetic Diversity Analysis. https://www.researchgate.net/profile/Andrea-Hoshino/publication/221925456_Microsatellites_as_Tools_for_Genetic_Diversity_Analysis/links/0d1c84f492b24ee605000000/Microsatellites-as-Tools-for-Genetic-Diversity-Analysis.pdf.

[38] Hedgecock D., Li G., Hubert S., Bucklin K., Ribes V. (2004). Widespread null alleles and poor cross-species amplification of microsatellite DNA loci cloned from the Pacific oyster, *Crassostrea gigas*. J. Shellfish Res..

[39] Hedgecock D., Launey S., Pudovkin A., Naciri Y., Lapegue S., Bonhomme F. (2007). Small effective number of parents (Nb) inferred for a naturally spawned cohort of juvenile European flat oysters *Ostrea edulis*. Mar. Biol..

[40] VanTassel N.M., Morris T.J., Wilson C.G., Zanatta D.T. (2021). Genetic diversity maintained in comparison of captive-propagated and wild populations of *Lampsilis fasciola* and *Ptychobranchus fasciolaris* (Bivalvia: Unionidae). Can. J. Fish. Aquat..

[41] Mathias P.T., Hoffman J.R., Wilson C.C., Zanatta D.T. (2018). Signature of postglacial colonization on contemporary genetic structure and diversity of *Quadrula quadrula* (Bivalvia: Unionidae). Hydrobiologia.

[42] Chacón G.M., Arias-Pérez A., Freire R., Martínez L., Ojea J., Insua A. (2021). Genetic characterization of wild, broodstock and seed samples of P*olititapes rhomboides* (Bivalvia: Veneridae): Implications for hatchery seed production. Aquac. Rep..

[43] Plough L.V., Hedgecock D. (2011). Quantitative trait locus analysis of stage-specific inbreeding depression in the Pacific oyster *Crassostrea gigas*. Genetics.

[44] Plough L., Shin G., Hedgecock D. (2016). Genetic inviability is a major driver of type III survivorship in experimental families of a highly fecund marine bivalve. Mol. Ecol..

[45] Lallias D., Taris N., Boudry P., Bonhomme F., Lapegue S. (2010). Variance in the reproductive success of flat oyster *Ostrea edulis* L. assessed by parentage analyses in natural and experimental conditions. Genet. Res..

[46] Mackintosh A., Laetsch D.R., Hayward A., Charlesworth B., Waterfall M., Vila R., Lohse K. (2019). The determinants of genetic diversity in butterflies. Nat. Commun..

[47] Cordero D., Delgado M., Liu B., Ruesink J., Saavedra C. (2017). Population genetics of the Manila clam (*Ruditapes philippinarum*) introduced in North America and Europe. Sci. Rep..

[48] Teixeira J.C., Huber C.D. (2021). The inflated significance of neutral genetic diversity in conservation genetics. Proc. Natl. Acad. Sci. USA.

[49] Ridlon A.D., Wasson K., Waters T., Adams J., Donatuto J., Fleener G., Froehlich H., Govender R., Kornbluth A., Lorda J. (2021). Conservation aquaculture as a tool for imperiled marine species: Evaluation of opportunities and risks for Olympia oysters, *Ostrea lurida*. PLoS ONE.

[50] Wang J., Santiago E., Caballero A. (2016). Prediction and estimation of effective population size. Heredity.

[51] Waples R.S., Hindar K., Karlsson S., Hard J.J. (2016). Evaluating the Ryman–Laikre effect for marine stock enhancement and aquaculture. Curr. Zool..

[52] Christie M.R., Marine M., French R., Waples R.S., Blouin M. (2012). Effective size of a wild salmonid population is greatly reduced by hatchery supplementation. Heredity.

[53] Morvezen R., Boudry P., Laroche J., Charrier G. (2016). Stock enhancement or sea ranching? Insights from monitoring the genetic diversity, relatedness and effective population size in a seeded great scallop population (*Pecten maximus*). Heredity.

[54] Baskett M.L., Burgess S.C., Waples R.S. (2013). Assessing strategies to minimize unintended fitness consequences of aquaculture on wild populations. Evol. Appl..

[55] Hold N., Murray L.G., Kaiser M.J., Hinz H., Beaumont A.R., Taylor M.I. (2013). Potential effects of stock enhancement with hatchery-reared seed on genetic diversity and effective population size. Can. J. Fish. Aquat. Sci..

[56] Frankham R., Ballou J., Briscoe D. (2002). Introduction to Conservation Genetics.

[57] Franklin I., Frankham R. (1998). How large must populations be to retain evolutionary potential?. Anim. Conserv. Forum.

[58] Waples R.S., Do C. (2010). Linkage disequilibrium estimates of contemporary Ne using highly variable genetic markers: A largely untapped resource for applied conservation and evolution. Evol. Appl..

[59] Lallias D., Boudry P., Lapegue S., King J.W., Beaumont A.R. (2010). Strategies for the retention of high genetic variability in European flat oyster (*Ostrea edulis*) restoration programmes. Conserv. Genet..

[60] Hedgecock D., Pudovkin A.I. (2011). Sweepstakes reproductive success in highly fecund marine fish and shellfish: A review and commentary. Bull. Mar. Sci..

[61] England P.R., Cornuet J.-M., Berthier P., Tallmon D.A., Luikart G. (2006). Estimating effective population size from linkage disequilibrium: Severe bias in small samples. Conserv. Genet..

[62] Waples R.S. (2006). A bias correction for estimates of effective population size based on linkage disequilibrium at unlinked gene loci. Conserv. Genet..

