# Supplementary material for: Population Genetics of Manila Clam (Ruditapes philippinarum) in China Inferred from Microsatellite Markers
Source: Biology (Basel). 2023 Apr 6;12(4):557. doi: 10.3390/biology12040557 (PMC10135866; doi:10.3390/biology12040557)
Supplement: Supplementary file 1 [file biology-12-00557-s001.zip › biology-2260263-supplementary.pdf]

biology

MDPI

Supplementary Materials

Population Genetics of Manila Clam (*Ruditapes philippinarum*) in China Inferred from Microsatellite Markers

Sichen Zheng, Tianshi Zhang, Kang Tu, Li Li, Zhihong Liu, Biao Wu, Liqing Zhou and Xiujun Sun

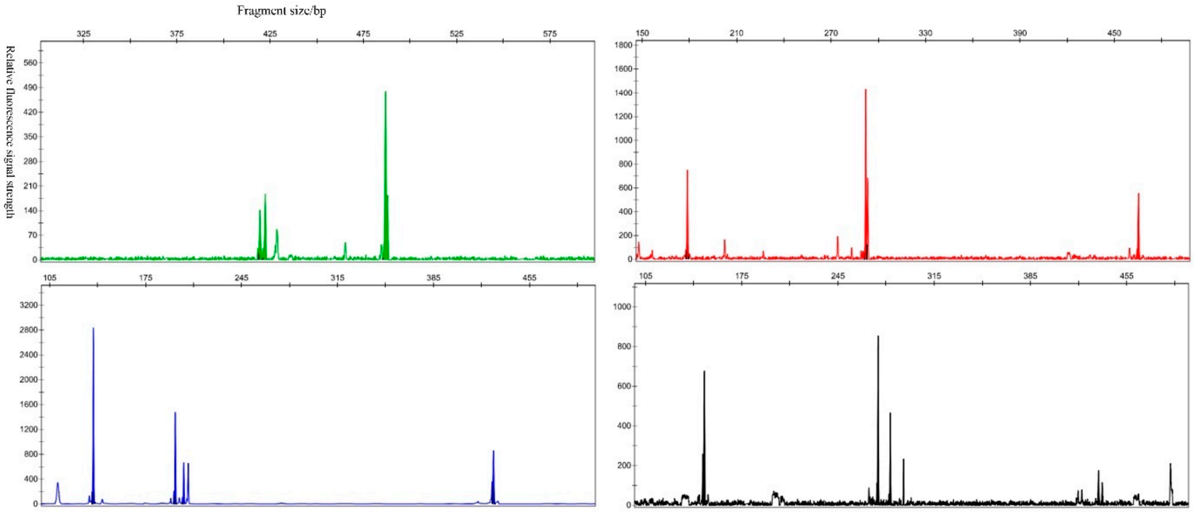

Figure S1. SSR profiles generated on a capillary sequencer for a single sample. Green color, Hex fluorescence; Red color, Rox fluorescence; Blue color, Fam fluorescence; Black color, Tamra fluorescence.

biology

MDPI

Table S1. Genetic diversity parameters of thirteen *R. philippinarum* populations.

Locus	Parameter	CZ	LZ	LJ	NB	SY	RZ	ZZ	BH	QD	HY	DG	ZH	XY	Total
Rpt83	N	6	7	8	9	5	8	6	5	5	5	6	7	10	14
	A_r	3.6	4.3	4.3	4.5	3.3	4.3	3.6	3.1	3.5	4.1	4.6	4.1	5.5	5.2
	G_D	0.596	0.774	0.647	0.753	0.633	0.727	0.621	0.505	0.610	0.741	0.817	0.709	0.848	
	H_o	0.419	0.333	0.138	0.250	0.286	0.414	0.292	0.167	0.286	0.545	0.385	0.087	0.353	0.304
	H_e	0.593	0.766	0.638	0.744	0.625	0.721	0.614	0.495	0.602	0.732	0.800	0.696	0.840	0.682
	F_{is}	0.296	0.569*	0.787*	0.668*	0.549*	0.431*	0.531*	0.670*	0.531*	0.264	0.529*	0.877*	0.584*	
	P	0.0944	0.0000*	0.0000*	0.0000*	0.0322	0.0000*	0.0000*	0.0048	0.0029*	0.1461	0.1229	0.0000*	0.0000*	
Rpt32	N	6	4	6	7	6	5	7	8	6	7	4	4	6	9
	A_r	4.1	3.0	4.2	3.9	3.8	2.6	3.8	4.0	3.4	4.2	3.2	2.7	3.7	4.2
	G_D	0.748	0.514	0.748	0.687	0.703	0.539	0.693	0.713	0.664	0.702	0.599	0.584	0.698	
	H_o	0.464	0.563	0.467	0.500	0.533	0.400	0.677	0.571	0.276	0.632	0.250	0.296	0.333	0.459
	H_e	0.743	0.515	0.743	0.684	0.701	0.536	0.693	0.710	0.658	0.700	0.590	0.579	0.692	0.657
	F_{is}	0.379*	-0.094	0.376*	0.272	0.242	0.257	0.022	0.199	0.585*	0.100	0.582*	0.493*	0.522*	
	P	0.0000*	0.3250	0.0002*	0.0002*	0.3978	0.6469	0.3929	0.5102	0.0021*	0.0019*	0.0012*	0.0763	0.0000*	
Rpt67	N	5	8	6	6	6	8	6	9	5	6	5	9	5	14
	A_r	3.4	4.8	3.6	3.4	3.2	4.5	3.2	4.1	3.3	3.9	3.0	4.5	3.6	4.1
	G_D	0.660	0.790	0.682	0.654	0.575	0.760	0.634	0.721	0.643	0.715	0.596	0.759	0.692	
	H_o	0.407	0.419	0.429	0.500	0.433	0.464	0.484	0.259	0.542	0.667	0.444	0.538	0.306	0.453
	H_e	0.655	0.784	0.677	0.651	0.573	0.755	0.631	0.712	0.641	0.714	0.592	0.755	0.686	0.679
	F_{is}	0.382*	0.469*	0.371*	0.236	0.247	0.389*	0.237	0.640*	0.158	0.068	0.255	0.291	0.558*	
	P	0.0000*	0.0000*	0.0001*	0.0022*	0.0012*	0.0540	0.0002*	0.0000*	0.0973	0.8358	0.0294	0.0007*	0.0000*	
Table 3. (continuation)															
Rpt105	N	9	8	7	9	6	5	5	6	4	8	4	5	7	16
	A_r	4.5	4.0	3.5	4.3	3.8	3.5	3.3	3.4	3.1	4.0	2.6	3.2	4.0	3.8
	G_D	0.728	0.662	0.573	0.708	0.635	0.641	0.641	0.647	0.586	0.675	0.521	0.548	0.732	
	H_o	0.219	0.484	0.531	0.345	0.483	0.467	0.677	0.500	0.333	0.519	0.238	0.333	0.200	0.410
	H_e	0.720	0.659	0.572	0.701	0.633	0.638	0.641	0.644	0.580	0.672	0.515	0.544	0.723	0.634
	F_{is}	0.699*	0.269	0.073	0.513*	0.240	0.272	-0.057	0.227	0.431	0.232	0.543*	0.392*	0.727*	
	P	0.0000*	0.0002*	0.4239	0.0001*	0.0959	0.0108	0.2383	0.0009*	0.0005*	0.0347	0.0002*	0.0040*	0.0000*	

Rpt106	N	5	6	5	6	7	6	6	7	4	7	6	6	8	14
	A_r	3.7	3.4	3.5	4.3	4.6	3.8	3.4	3.9	4.0	4.8	4.6	4.4	4.8	5.5
	G_D	0.696	0.580	0.678	0.708	0.796	0.726	0.662	0.650	0.900	0.816	0.814	0.758	0.798	
	H_o	0.594	0.156	0.065	0.069	0.387	0.464	0.107	0.103	0.000	0.412	0.417	0.250	0.231	0.250
	H_e	0.694	0.573	0.668	0.696	0.790	0.721	0.652	0.640	0.800	0.804	0.797	0.745	0.787	0.721
	F_{is}	0.147	0.731*	0.905*	0.903*	0.514*	0.360*	0.838*	0.841*	1.000*	0.495*	0.488*	0.670*	0.711*	
	P	0.6132	0.0000*	0.0000*	0.0000*	0.0000*	0.0000*	0.0000*	0.0000*	0.0203	0.0001*	0.1664	0.0000*	0.0000*	
Rpt100	N	5	6	4	4	6	6	5	3	5	4	6	6	6	9
	A_r	3.2	3.2	3.2	3.2	3.4	2.7	2.5	2.3	2.0	3.1	2.5	3.1	3.8	3.2
	G_D	0.567	0.469	0.585	0.531	0.584	0.359	0.416	0.335	0.196	0.511	0.305	0.526	0.660	
	H_o	0.483	0.563	0.645	0.581	0.656	0.375	0.516	0.387	0.207	0.643	0.292	0.357	0.816	0.502
	H_e	0.566	0.471	0.586	0.531	0.585	0.359	0.417	0.336	0.196	0.514	0.305	0.523	0.662	0.465
	F_{is}	0.149	-0.199	-0.102	-0.094	-0.124	-0.045	-0.242	-0.156	-0.057	-0.257	0.045	0.321	-0.235	
	P	0.0001*	0.9929	0.1316	0.7140	0.9904	0.9998	0.9579	0.6181	1.0000	0.3923	0.0000*	0.0013*	0.7304	

Table 3. (continuation)

Rpt36	N	8	8	9	8	7	10	5	8	9	7	8	6	7	16
	A_r	4.3	4.9	4.5	4.9	4.4	5.1	3.5	4.6	4.9	4.8	3.7	4.0	4.6	4.7
	G_D	0.716	0.814	0.769	0.826	0.767	0.820	0.667	0.800	0.780	0.805	0.680	0.695	0.796	
	H_o	0.516	0.469	0.688	0.594	0.625	0.625	0.906	0.594	0.828	0.679	0.875	0.724	0.525	0.665
	H_e	0.713	0.809	0.768	0.822	0.765	0.816	0.671	0.797	0.780	0.803	0.684	0.695	0.792	0.763
	F_{is}	0.279*	0.424*	0.106	0.281*	0.185	0.237	-0.358	0.258	-0.062	0.157	-0.286	-0.043	0.340*	
	P	0.3037	0.0002*	0.1909	0.1130	0.4389	0.0006	0.0275	0.0500	0.0005*	0.0005	0.0000*	0.8536	0.0000*	
Rp-03	N	9	8	7	7	8	7	7	11	10	6	6	2	7	18
	A_r	5.1	4.7	4.5	4.8	4.8	4.0	4.3	4.8	4.7	3.5	3.9	2.0	4.3	4.5
	G_D	0.811	0.778	0.787	0.806	0.772	0.719	0.712	0.772	0.762	0.665	0.711	0.500	0.766	
	H_o	0.531	0.516	0.567	0.500	0.813	0.656	0.906	0.875	0.897	0.750	0.958	0.966	0.775	0.747
	H_e	0.807	0.774	0.784	0.802	0.773	0.718	0.715	0.774	0.765	0.666	0.716	0.508	0.766	0.736
	F_{is}	0.345*	0.337*	0.280*	0.380*	-0.052	0.088	-0.272	-0.133	-0.176	-0.128	-0.348	-0.931	-0.011	
	P	0.0000*	0.0000*	0.0242	0.0000*	0.0003*	0.3837	0.4073	0.9956	0.9160	0.2140	0.0300	0.0000*	0.0000*	
Asari16	N	4	9	6	3	7	6	8	3	4	3	4	5	5	16
	A_r	2.2	5.2	2.5	1.7	3.1	3.2	2.9	1.7	1.7	2.3	2.6	3.0	2.6	3.3
	G_D	0.310	0.823	0.315	0.149	0.451	0.604	0.384	0.153	0.135	0.528	0.405	0.602	0.370	
	H_o	0.355	0.719	0.188	0.094	0.406	0.781	0.438	0.161	0.069	0.821	0.391	1.000	0.303	0.440

	H_e	0.310	0.821	0.313	0.148	0.450	0.607	0.384	0.153	0.134	0.533	0.405	0.609	0.369	0.403
	F_{is}	-0.146	0.126	0.404*	0.372	0.098	-0.293	-0.141	-0.056	0.489	-0.556	0.034	-0.662	0.182	
	P	0.9632	0.0070	0.0019*	0.0720	0.0007*	0.0000	1.0000	0.9711	0.0001*	0.0000*	0.4281	0.0012*	0.0000*	

Table 3. (continuation)

Rp-07	N	5	5	6	5	6	5	5	4	4	4	5	3	5	12
	A_r	3.2	2.7	3.3	3.3	2.9	2.4	3.1	2.6	2.6	2.6	2.6	2.4	2.9	3.2
	G_D	0.552	0.385	0.477	0.553	0.442	0.400	0.498	0.540	0.567	0.570	0.473	0.445	0.585	
	H_o	0.438	0.125	0.250	0.688	0.469	0.500	0.625	0.719	0.931	0.857	0.500	0.586	0.550	0.557
	H_e	0.550	0.381	0.474	0.556	0.442	0.402	0.500	0.543	0.574	0.575	0.473	0.447	0.584	0.500
	F_{is}	0.207	0.675*	0.476*	-0.242	-0.060	-0.249	-0.255	-0.330	-0.642	-0.053	-0.057	-0.319	0.060	
	P	0.0000*	0.0000*	0.0000*	0.5222	0.9998	0.9652	0.7615	0.0000*	0.0011*	0.0001*	0.0000*	0.1728	0.0243	
Rpt23	N	9	7	7	6	9	8	5	7	6	6	6	3	6	12
	A_r	5.0	4.7	4.6	4.5	5.1	4.7	3.9	4.1	3.9	3.7	4.3	3.4	4.6	4.7
	G_D	0.811	0.801	0.800	0.791	0.820	0.805	0.721	0.748	0.720	0.722	0.750	0.586	0.801	
	H_o	0.889	0.500	0.844	0.750	0.875	0.781	0.938	0.781	0.931	0.929	0.625	0.679	0.850	0.798
	H_e	0.813	0.797	0.801	0.790	0.821	0.805	0.725	0.749	0.724	0.725	0.747	0.588	0.801	0.760
	F_{is}	-0.096	0.376*	-0.055	0.052	-0.067	0.030	-0.300	-0.044	-0.293	-0.287	0.167	-0.158	-0.062	
	P	0.0416	0.0041*	0.9197	0.6149	0.4788	0.8183	0.0011*	0.3589	0.0109	0.3242	0.1659	0.0002*	0.3430	
All	N	6.5	6.9	6.5	6.4	6.6	6.7	5.9	6.5	5.6	5.7	5.5	5.1	6.5	
	A_r	3.8	4.1	3.8	3.9	3.8	3.7	3.4	3.5	3.4	3.7	3.4	3.3	4	
	G_D	0.654	0.672	0.642	0.651	0.653	0.645	0.604	0.599	0.597	0.677	0.606	0.61	0.704	
	H_o	0.483	0.441	0.437	0.443	0.542	0.539	0.597	0.465	0.482	0.678	0.489	0.529	0.476	0.508
	H_e	0.651	0.668	0.639	0.648	0.651	0.644	0.604	0.596	0.587	0.676	0.602	0.608	0.700	0.636
	F_{is}	0.261	0.344	0.319	0.320	0.169	0.165	0.012	0.223	0.193	0.000	0.194	0.133	0.323	

Note: N, number of alleles; A_r, allelic richness; G_D, gene diversity; H_o, the observed heterozygosity; H_e, the expected heterozygosity; F_{is}, inbreeding coefficient, "*" indicates significant departure from Hardy-Weinberg equilibrium after Bonferroni correction ($P < 0.005$).

Table S2. Genetic differentiations and gene flow of thirteen *R. philippinarum* populations at different loci

Locus	F_{st}	F_{it}	F_{is}	N_m
Rpt83	0.1443	0.6328	0.5708	1.4821
Rpt32	0.0990	0.3720	0.3031	2.2765
Rpt67	0.0412	0.3725	0.3455	5.8168
Rpt105	0.0360	0.3780	0.3547	6.6869
Rpt106	0.0565	0.6614	0.6411	4.1745
Rpt100	0.0398	-0.0444	-0.0877	6.0309
Rpt36	0.0357	0.1741	0.1434	6.7442
Rp-03	0.0394	0.0354	-0.0042	6.0936
Asari16	0.1729	0.1013	-0.0865	1.1958
Rp-07	0.0651	-0.0303	-0.1021	3.5887
Rpt23	0.0376	-0.0096	-0.0491	6.3996
ALL	0.0663	0.2396	0.1855	4.5900

$N_m = 0.25\times(1–F_{st})/F_{st}$
